# From Exit to Entry: Long-term Survival and Transmission of *Salmonella*

**DOI:** 10.3390/pathogens1020128

**Published:** 2012-10-24

**Authors:** Landon L. Waldner, Keith D. MacKenzie, Wolfgang Köster,, Aaron P. White

**Affiliations:** Vaccine and Infectious Disease Organization, University of Saskatchewan, Saskatoon, Saskatchewan, S7N 5E3, Canada. E-Mails: llw310@mail.usask.ca (L.L.W); keith.mackenzie@usask.ca (K.D.M.); wolfgang.koester@usask.ca (W.K.)

**Keywords:** *Salmonella*, transmission, persistence, outbreak, rdar morphotype, tomato, sprouts, chocolate, genomics

## Abstract

*Salmonella* spp. are a leading cause of human infectious disease worldwide and pose a serious health concern. While we have an improving understanding of pathogenesis and the host-pathogen interactions underlying the infection process, comparatively little is known about the survival of pathogenic *Salmonella* outside their hosts. This review focuses on three areas: (1) *in vitro* evidence that *Salmonella* spp. can survive for long periods of time under harsh conditions; (2) observations and conclusions about *Salmonella* persistence obtained from human outbreaks; and (3) new information revealed by genomic- and population-based studies of *Salmonella* and related enteric pathogens. We highlight the mechanisms of *Salmonella* persistence and transmission as an essential part of their lifecycle and a prerequisite for their evolutionary success as human pathogens.

## 1. Introduction

The evolutionary success of bacterial pathogens is dependent on their ability to colonize and cause disease in susceptible hosts. Equally important is how effectively these pathogens are transmitted between hosts. For human-specific or human-adapted pathogens, such as *Helicobacter*, *Neisseria* species and others, it is assumed that life outside the host represents only a small part of their lifecycle [1]. Many human commensal bacteria, such as *Streptococcus* and *Staphylococcus* spp., have similar host-restricted lifestyles, although there are examples of commensals, such as *Escherichia coli*, that survive both inside and outside of the host [2,3]. Enteric bacterial pathogens are typically not host-restricted and have a cyclical lifestyle, with mechanisms for inducing passage out of the host and adaptations for prolonged survival in the environment [4]. Zoonotic transmission of enteric pathogens is also common, such as the acquisition of *Campylobacter* infections from chickens and enterohemorrhagic *E. coli* (EHEC) from cattle. This review is focused on the transmission etiology of *Salmonella* spp., one of the world’s most important enteric pathogens. We describe possible mechanisms that enable *Salmonella* spp. to persist in non-host environments and hypothesize how this contributes to *Salmonella* transmission in today’s industrialized world. 

### 1.1. Salmonella Taxonomy and Human Disease

The genus *Salmonella* is comprised of two species, *S. enterica* and *S. bongori*, with *S. enterica* being divided into six subgroups (*enterica*, *salamae*, *arizonae*, *diarizonae*, *indica*, and *houtenae*). *S. enterica* subspecies *enterica* strains primarily infect warm-blooded hosts and are responsible for >95% of human infections, while the remaining five subspecies and *S. bongori* primarily infect cold-blooded hosts. The division of *Salmonella* into only two species is based on DNA-DNA hybridization and the observed high genetic relatedness between strains [5,6]. Current taxonomy is largely based on the White-Kauffmann-Le Minor scheme that uses serotyping to classify *Salmonella* strains isolated from human patients; each unique combination of flagellar, lipopolysaccharide (LPS) and capsular antigen reactivity results in the designation of a new serovar [7]. There are now >2600 serovars, and ~60% are members of *S. enterica* subspecies *enterica*. For the remainder of this review, all serovars will be listed in their shortened form (*i.e.*, *Salmonella *ser. Typhimuriumfor *S. enterica* subsp. *enterica* serovar Typhimurium).

Most cases of human disease are caused by serovars of *S. enterica* subsp. *enterica*. Numerous serovars can cause gastroenteritis, and they are collectively referred to as nontyphoidal Salmonellae (NTS). The most common NTS serovars worldwide are Typhimurium and Enteritidis [8]. Serovars Typhi and Paratyphi A may cause blood stream invasion in the absence of gastroenteritis and are referred to as Invasive Salmonellae; the disease they cause is usually classified as typhoid fever. Recently, the distinction between serovars has been blurred by an appreciation of an MLST type of *Salmonella* ser. Typhimurium ST313 [9] that is the most common cause of blood stream infection (frequently without gastroenteritis) in Sub Saharan Africa [10]. NTS in both the developed world and especially in Africa cause sepsis and death in immune suppressed patients. NTS may also cause death by dehydration in children or susceptible adults. The current worldwide estimates are 94 million cases of *Salmonella* gastroenteritis annually with 150,000 deaths [11], and 21 million cases of typhoid fever with approximately 200,000 deaths [12]. Within North America, incidences of typhoid fever are treated with antibiotics, and gastroenteritis is typically self-limiting. However, the economic costs of *Salmonella *infections, including both medical care and lost productivity, have been estimated in the billions of dollars [13]. This review will focus primarily on NTS serovars that cause gastroenteritis with discussion of serovars Typhi or Paratyphi only for comparison purposes.

*S. enterica *subsp. *enterica* serovars can also be described as either host-generalist, host-adapted or host-restricted [14]. These categories have major implications on the transmission characteristics of each isolate, which will be described later. Host-adapted or -restricted serovars have evolved strategies for persisting inside of the host and evading immune defenses. *Salmonella* ser. Typhi, for example, disseminates from the gastrointestinal tract to the reticuloendothelial system, where it can colonize the surface of gallstones [15]. Approximately 1–6% of patients who have been infected with *Salmonella* ser. Typhi become chronic, asymptomatic carriers [16,17,18]. In contrast, pathogenesis of host-generalist serovars usually leads to gastroenteritis, and infected patients shed *Salmonella* for a relatively short period of time. There have been instances where shedding occurs after recovery, but only at low levels [19]. The lifecycle of host-generalist NTS strains has a greater dependency on survival in the environment, presumably due to their reduced long-term shedding capacity. 

## 2. Results and Discussion

### 2.1. In vitro Evidence that Salmonella spp. Can Survive for Long Periods of Time under Harsh Conditions

*Salmonella* spp. are known to survive in non-host environments [20], but the mechanisms of persistence are not well understood. For example, the well-characterized acid tolerance response [21] is usually presumed to be a pathogenesis adaptation to ensure smooth passage of *Salmonella* through the mammalian stomach. From analysis of *Salmonella* persistence in poultry houses and other food processing environments, the idea took hold that vectors (*i.e.*, rodents, insects) represent a main environmental reservoir of *Salmonella* spp. [22,23]. More recently, there is evidence of biofilm formation, a multicellular behavior that may enable *Salmonella* spp. to survive long-term in the environment without requiring an animal reservoir. 

Fimbriae (or pili) have long been thought to play a central role in the interactions between bacterial pathogens and their hosts. Genome sequencing revealed that *S. enterica* isolates can possess at least 15 different fimbrial types [24]. Since most fimbrial operons had a scattered distribution within the *S. enterica* serovars [24,25], it was assumed that different fimbrial types were required for colonization of different hosts. Curli (or thin aggregative fimbriae) were distinct in that their subunit genes were detected throughout *S. enterica* subsp. *enterica* (*i.e.*, 603 of 604 isolates, representing 95 serovars) [26] and even *E. coli* [25]. Biochemical characterization of curli fibers showed they are resistant to boiling, bases, detergents and proteolytic digestion [27]. The presence of these incredibly resistant structures on the cell surface was hypothesized to be a potential survival advantage for *Salmonella* during passage through the mammalian stomach into the small intestine [27]. The conservation of curli throughout *S. enterica *indicated that these organelles have an important evolutionary role in the *Salmonella* lifecycle.

Curli production was associated with cell-cell aggregation and the formation of adhesive colonies by both *S. enterica* and *E. coli* isolates [28,29]. Ute Romling and colleagues [30] termed this phenotype the “rdar morphotype” for *r*ed, *d*ry, and *r*ough colonies formed by *Salmonella* ser. Typhimurium on nutrient-limited laboratory media containing the indicator dye Congo red. Romling *et al*. also demonstrated that the curli genes (*csgDEFG* and *csgBAC*) were functionally interchangeable between *Salmonella* and *E. coli* [31]. Further characterization of the rdar morphotype led to the discovery that cellulose was an integral part of the extracellular matrix, tightly linked to curli on the cell surface [32], and responsible for “long-range” interactions within rdar colonies [33]. This allows the entire colony to be lifted off the agar surface in one piece [30]. The chemical resistance and strength of cellulose and curli suggests that they may function as an inert matrix or scaffolding that holds cells together. Additional components of the rdar matrix have since been discovered: an O-antigen capsule [34] and additional polysaccharides [34,35]; as well as a large, cell surface protein termed BapA that contains repetitive stretches of amino acids that are presumed to be involved in aggregation [36]. The subsequent discovery that curli fibers represent a “functional” amyloid [37,38] and that amyloid fimbrial structures can be found in diverse natural biofilms [39] is suggestive that the rdar morphotype represents a biofilm-like state for *Salmonella*. 

The resistance properties conferred by the rdar morphotype suggest that this physiology may have a role in long-term survival. Rdar morphotype cells have shown increased resistance to hydrogen peroxide and acid [40], sodium hypochlorite [41,42,43] and various disinfecting agents [44,45], as well as an increased ability to stick to abiotic surfaces [46,47]. We performed some of the first *in vitro* experiments to compare survival of rdar-forming (rdar^+^) *Salmonella* ser. Typhimurium to isogenic mutants that were lacking different extracellular components [43]. Rdar^+^ cells survived significantly better than mutants under desiccation and starvation conditions [43], and the O-Ag capsule appeared to be critically important for this survival [34]. After 14 months stored on plastic, cell numbers in rdar colonies were at 2–5% of starting CFU levels [43], and cells remained at this level even after 30 months [48], suggesting that survival could go on indefinitely. These 30-month-old rdar^+^ cells were still able to cause infection in mice [48]. The ability of rdar^+ ^cells to persist and remain pathogenic in this physiological state after such a long period of time was unexpected. This strongly suggested that *S. enterica* isolates do not need an animal reservoir to survive long-term in the environment. One consistent finding to date is that the rdar morphotype has not been associated with increased virulence [42,49]. Decreased invasion of epithelial cell lines was recently reported for another biofilm-like state of serovar Typhimurium [50], suggesting this may be a common theme. However, survival and persistence are as equally important as pathogenic ability if *S. enterica* isolates have an extended phase of life outside their hosts.

The evidence accumulated so far indicates that the rdar morphotype represents a conserved survival strategy for *S. enterica*. Metabolomic and transcriptional analysis has shown that *Salmonella* ser. Typhimurium rdar^+^ cells have up-regulated several well-known stress resistance pathways, such as reactive oxygen species defense, osmoprotection and nutrient acquisition [51]. The changes in metabolism required for extracellular matrix production were synchronized with up-regulation of the resistance adaptations, suggesting that there is a coordinated shift in physiology as cells enter this state [51]. Grantcharova *et al*. [52] analyzed three different *Salmonella* biofilm models related to the rdar morphotype and observed that there was always a balance between multicellular aggregates (*i.e.*, rdar morphotype) and planktonic cells. The curli-related transcriptional binding protein CsgD was found to act as a bistable control switch between these two cell populations [52]. Interestingly, bistable switches often play key roles in high investment processes, such as cellular differentiation, in which only the end-result of the process is functional [53]. In the case of *S. enterica*, this type of control strategy would maintain the developmental potential of cell populations and maximize the chances for survival in many natural environments.

#### 2.1.1. Does *Salmonella* Enter a Viable, Non-culturable State?

**Figure 1:**
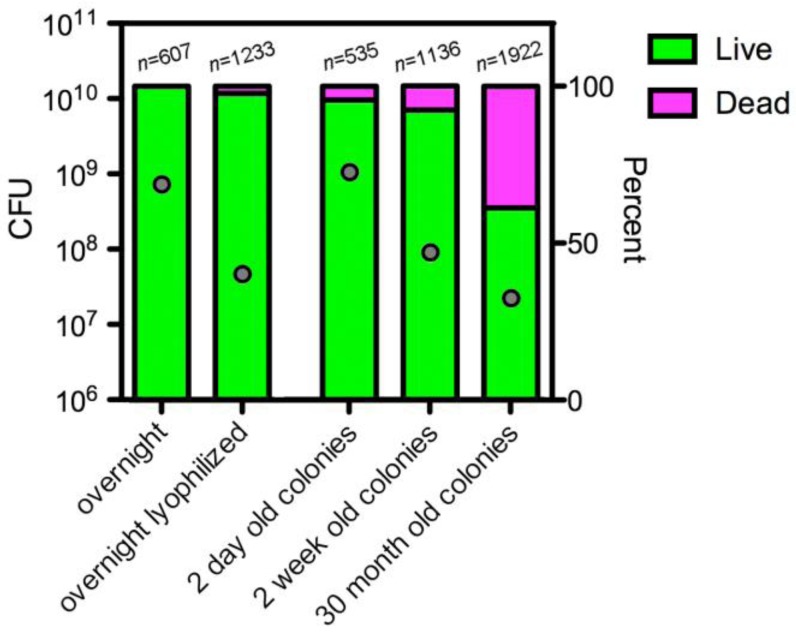
Survival of *Salmonella* ser. Typhimurium in Rdar Morphotype Colonies Compared to Liquid Cultures. Dots represent the total CFU from 1 mL aliquots of cells grown for 18 h in Luria broth at 37 °C (overnight) or rdar morphotype colonies grown on 1% tryptone agar for two days at 28 °C. To ensure consistency, overnight cultures were normalized to an optical density of 1.0 at 600 nm prior to removing aliquots. The “overnight lyophilized” samples represent 1 mL aliquots of cells that were frozen and lyophilized for 48 h prior to measurement. Rdar morphotype colonies were stored for two weeks or 30 months, one colony per well, in a plastic, 24-well tissue culture plate [43] prior to measurement. Each grey dot represents the average CFU value from at least four biological replicates. Cells samples were stained using a Live/Dead bacterial viability kit (Invitrogen, Carlsbad, CA, USA, cat. No. L-7012) and enumerated by manual scanning on a fluorescent microscope; *n* represents the total number of cells that were counted from two to three biological replicates of each sample type. The bars reflect the percentage of cells that were scored as live (green) or dead (magenta), with a combined total of 100%.

The long-term survival of *Salmonella* ser. Typhimurium cells in rdar morphotype colonies suggested that the cells could be in a metabolically dormant state, perhaps similar to persister cells within biofilms [54]. After 30 months storage on plastic, rdar morphotype cells were hypersensitive to bile salts [48], indicating that the cell membranes may be damaged [55]. However, while the total CFU count was <5% of the starting value after 30 months, greater than 50% of the cells were scored as alive after staining with a Live/Dead bacterial viability kit (Invitrogen, Carlsbad, CA, USA, cat. No. L-7012) (Figure 1). The physiological state of these cells was not further characterized, but it is possible that they could represent viable, non-culturable cells (VBNC). If so, this would imply that long-term survival of *Salmonella* was underestimated in our *in vitro* experiments [43,48]. 

The pathological significance of the VBNC phenotype in the *S. enterica *lifestyle is uncertain. Colwell *et al*. [56] first proposed the idea of VBNC *Salmonella* in 1984 after monitoring the status of *Salmonella* ser. Enteritidis cells suspended in river water. These cells became non-culturable as early as 48 hours and could be resuscitated by the addition of nutrients. It is thought that the VBNC state may represent either the crippling effect of extreme stress or a regulated *Salmonella* survival mechanism. Since *Salmonella* is a foodborne pathogen, there is considerable public health concern whether VBNC can retain growth or pathogenic potential once introduced to new surroundings. *Salmonella* VBNC cells can be resuscitated [57,58,59,60], suggesting that they could potentially grow and re-infect. However, in several experimental models, the *Salmonella* VBNC cells were unable to cause infections in chickens or mice, indicating that cells failed to resuscitate during passage through the gastrointestinal tract [61,62,63]. Nevertheless, since VBNC cells are proposed to play a role in transmission and survival of other enteric pathogens, such as *Vibrio cholerae* [64], they could play a similar role for *S. enterica*. 

### 2.2. Salmonella in the Environment: Lessons from Outbreaks

*Salmonella* serovars cause an estimated 1.2 million illnesses annually in the U. S. and are the most common causes of hospitalization and death among foodborne pathogens that are tracked by the Foodborne Diseases Active Surveillance Network (FoodNet) [65]. Despite the efforts of highly developed regulatory bodies like the Food and Drug Administration (FDA) and United States Department of Agriculture (USDA), the incidence of *Salmonella* was 3% higher in 2010 than it was in 1996-1998. This is in contrast to other foodborne pathogens such as EHEC, *Campylobacter*, *Listeria*, *Shigella* and *Yersinia* spp., whose occurrences have decreased by 44%, 27%, 38%, 57% and 52%, respectively [65]. The variety of different foods that *Salmonella* can be isolated from is a testament to its widespread presence in the food supply chain. *Salmonella* outbreaks have been linked to contaminated meat, poultry, eggs, unpasteurized dairy products, tomatoes, sprouts, melons, lettuce, mangoes, chocolate, powdered infant formula, raw almonds, dry seasonings, cereals and peanut butter. Outbreaks of *S. enterica* associated with these food vehicles involve host-generalist, NTS serovars, as opposed to human-adapted serovar Typhi. In 2009, from 6,371 isolates that were serotyped by the U.S. Center for Disease Control and various State health departments, the percentages of the top five *S. enterica* serovars were Enteritidis (19.2%), Typhimurium (16.1%), Newport (12.1%), Javiana (8.5%), and Heidelberg (3.6%) [66]. 

A historical analysis of *Salmonella* outbreaks highlights the adaptability of this pathogen to a variety of different food processing environments. Environments, like pond water, the inside of a tomato fruit, stainless steel factory surfaces or inside low-moisture foods are so different that one may wonder how *Salmonella* is able to persist in such diverse settings. Table 1 describes selected outbreaks as far back as 1970 and was compiled with the objective of illustrating themes in *Salmonella* adaptability. 

#### 2.2.1. Tomato-related *Salmonella* Outbreaks

From 1990–2012, the total number of reported cases of *Salmonella* in the United States involving tomatoes as a food vehicle was 2,059. It is likely that this statistic vastly underestimates the impact of tomato-related *Salmonella* infections, as it is suggested that only one of every 38 cases is reported to public health authorities [13]. Several epidemiological studies have tried to pinpoint the source of contamination for these outbreaks. Table 1 shows that investigations of multi-state outbreaks often lead investigators to packing houses or to the produce fields that supply them. However, direct isolation of *Salmonella* from the production environment rarely occurs. For example, from all tomato-associated outbreaks listed in Table 1, in one instance only were investigators successful in obtaining the isolate responsible [69]. Nevertheless, investigators are increasingly able to use epidemiological evidence as a primary method for identifying the source(s) of contamination. 

**Table 1:** Selected Outbreaks of Human Gastroenteritis Caused by *S. enterica*.

Food Vehicle	*S. enterica* Serovar	Cases	Year	Traceback	Reference
Tomato	Javiana	176	1990	Packing plant	[67]
Tomato	Montevideo	100	1993	Packing plant	[67]
Tomato	Baildon	86	1998	Grower/Packing plant	[68]
Tomato	Newport	510	2002	Pond water at grower	[69]
Tomato	Braenderup	125	2004	Packing plant	[70,71]
Tomato	Multiserotype	561	2004	Packing plant	[70,72]
Tomato	Braenderup	82	2005	Grower	[73]
Tomato	Newport	72	2005	Pond water at grower	[69,73]
Tomato	Typhimurium	190	2006	Packing plant	[73,74]
Tomato	Newport	115	2006	Not stated	[73]
Alfalfa sprouts	Stanley	>242	1995	Alfalfa Seeds	[75]
Alfalfa sprouts	Newport	>133	1995	Alfalfa Seeds	[76]
Alfalfa sprouts	Montevideo	417	1996	Alfalfa Seeds	[77]
Alfalfa sprouts	Melegridis	75	1996	Alfalfa Seeds	[77]
Alfalfa/Clover	Senftenberg	60	1997	Clover Seeds	[77]
Alfalfa sprouts	Infantis/Anatum	109	1997	Alfalfa Seeds	[78]
Alfalfa sprouts	Havana	18	1998	Alfalfa Seeds	[77]
Alfalfa sprouts	Cubana	22	1998	Alfalfa Seeds	[77]
Alfalfa sprouts	Mbandaka	87	1999	Alfalfa Seeds	[79]
Alfalfa sprouts	Muenchen	157	1999	Alfalfa Seeds	[80]
Clover sprouts	Typhimurium	112	1999	Clover seeds	[81]
Alfalfa sprouts	Kottbus	31	2003	Alfalfa seeds	[82]
Alfalfa sprouts	Saintpaul	228	2009	Alfalfa Seeds	[83]
Alfalfa sprouts	Newport	44	2010	Not Stated	[84]
Alfalfa/Clover	Typhimuirum (I 4,[5],12:i:)	140	2010	Not stated	[85]
Alfalfa sprouts	Enteriditis	25	2011	Not stated	[86]
Paprika Chips	Saintpaul, Javiana, and Rubislaw	>670	1993	Paprika powder	[87]
Toasted Oat Cereal	Agona	209	1998	Air-handling systemand vitamin spray mixer	[88]
Squid/shrimp Crackers	Chester and Oranienberg	1634	1999	Unknown	[89,90]
Chocolate	Durham	110	1970	Cocoa powder	[91]
Chocolate	Eastbourne	217	1973	Cocoa bean	[92,93]
Chocolate	Napoli	245	1982	Not stated	[94]
Chocolate	Nima	24	1985	Not stated	[95]
Chocolate	Typhimurium	349	1987	Avian wildlife reservoir suspected	[96]
Chocolate	Oranienburg	439	2001	Unknown	[97]

In many tomato-associated *Salmonella* outbreaks, investigators concluded that *Salmonella* had been introduced to plants through contaminated water. Investigation of outbreaks in 1990 and 1993 (Table 1) suggested that improper monitoring of chlorination of a water bath at a packing plant lead to the cross-contamination of tomatoes [67]. In an outbreak in 2005, *Salmonella* ser. Newport was isolated from an irrigation pond next to the produce field [69]. This same strain of serovar Newport was also responsible for a large outbreak in 2002, suggesting a persistent contamination of the water source. In another study investigating tomato-producing farms in 2009-2010, local groundwater, irrigation pond water, pond sediment, irrigation ditch water, rhizosphere and irrigation ditch soil, leaves, tomatoes, harvest bins and sanitary facilities were tested for *Salmonella* [98]. Twenty-nine percent of farms were positive for *Salmonella*, with the conclusion being that irrigation water and soil led to pre-harvest contamination of tomatoes [98].

*Salmonella* has been isolated from streams and rivers where farmers obtain their irrigation water and is usually associated with fecal contamination [99,100,101,102]. Santo Domingo *et al*. [103] inoculated four strains of *Salmonella* ser. Typhimurium into water samples collected from the Great Miami River, which is an irrigation source for farmers in this region. As measured by culturability, *Salmonella* levels dropped from 10^8^ CFU/mL to 10^4^ CFU/mL after 45 days, whereas by direct microscope counts, the cell levels remained relatively constant. This discrepancy suggested that a large percentage of cells in the population were dead, however flow cytometry experiments using a live-dead stain demonstrated that most cells were viable [103]. It was suggested that these cells could represent a VBNC population. *Salmonella* may also persist in aqueous systems in the form of biofilms. Recently, *Salmonella* was isolated from natural biofilms in Spring Lake, San Marcos, Texas [104]. The authors of this study hypothesized that biofilms could facilitate the long-term persistence of *Salmonella* and allow for eventual transfer into the food chain when waters were tapped for irrigation.

Several studies have focused on contamination of tomato plants from *Salmonella*-containing irrigation water. Hintz *et al*. [105] explored the potential routes of tomato plant contamination using a clinical isolate of *Salmonella* ser. Newport. From 92 tomato plants irrigated with contaminated water, 25 were confirmed positive for serovar Newport. Sixty-five percent of the positive samples were contaminated in the roots, while the remainder of samples had *Salmonella* in the stems, leaves and fruits. Tomato fruit contamination was present but only accounted for 6% of the total contamination. High levels of root contamination suggest that this represents an important entry route for *Salmonella* into tomato plants. Guo *et al*. [106] performed a root-based invasion assay using five different *Salmonella* serovars involved in produce-related outbreaks. Tomato plants were grown hydroponically and exposed to a nutrient solution containing 10^4^-10^5^ CFU *Salmonella*/mL. Within one day of exposure, there were ~10^3^ CFU/g in the stems and seed leaves of germinating tomato seedlings [106]. In another study, *Salmonella* ser. Montevideo was internalized into tomato plants from contaminated irrigation water, but the fruit did not show internalization [107]. Gu *et al*. [108] used confocal microscopy to monitor the spread of *Salmonella* in tomato plants after internalization. In parts of the plant that were directly inoculated, *Salmonella* was observed in both vascular components of the plant, the phloem and xylem. In adjacent parts of the plant that were not directly inoculated, *Salmonella* was only observed in the phloem, suggesting that colonization of other parts of the tomato plant occurs via the phloem. One caveat for many of these *Salmonella* internalization studies is that high inocula (*i.e.*, 10^7^–10^8^ CFU/mL) were often used, which is likely unrealistic in nature. 

The probability of *Salmonella* internalization into the tomato fruit is far greater in the harvest and post-harvest stage. In these stages, the tomato surface is more likely to be damaged, allowing access of *Salmonella* that has contaminated the surface to the insides of the tomato. Packing plants often have communal water baths where tomatoes are washed and disinfected. If these water baths contain cold water contaminated with *Salmonella*, the water is taken up and the tomatoes can easily become contaminated [109]. To reduce the chances of internalization, current FDA guidelines recommend maintaining water temperature at least 10°F warmer than pulp temperature (U.S. FDA). It has also been observed that *Salmonella* cell numbers within chopped tomatoes can increase by 1.5 to 2.5 log units after only 24-hours storage [109]. 

In summary, analysis of tomato-associated *S. enterica* outbreaks indicate that the bacterium can survive for long periods of time in the environment whether it be in water or on the surfaces of plants. Furthermore, different NTS serovars may gain entry to the tomato fruit during processing and have the capacity to reach high cell numbers. In addition to the larger outbreaks we have described, presumably there are also many sporadic cases of *Salmonella* infection associated with ingestion of contaminated tomatoes. 

#### 2.2.2. Sprout-related *Salmonella* Outbreaks

During the past 15–20 years, over twenty outbreaks of *Salmonella* gastroenteritis have occurred due to the ingestion of contaminated alfalfa and clover sprouts (the smallest outbreak we make reference to in Table 1 had 18 confirmed cases of human infection). Epidemiological analysis has shown that contaminated seeds are the major source of contamination in most sprout-related outbreaks. In 1995, an outbreak of *Salmonella* ser. Stanley affecting people in the US and Finland was traced back to contaminated alfalfa grown by nine different sprout growers who were supplied by a single Dutch seed shipper [75]. In late 1995 and 1996, an outbreak of *Salmonella* ser. Newport affecting people in Oregon and British Columbia was traced back to contaminated seeds supplied by a different Dutch shipping company [76]. In a 1997 outbreak, alfalfa seeds that tested positive for *Salmonella* ser. Anatum were received from local farms in Kansas and Missouri [78]. In a 1998 outbreak of serovars Havana and Cubana, *S. enterica* strains isolated from seeds had pulsed-field gel electrophoresis patterns identical to those isolated from infected patients [110]. The fact that alfalfa seeds are often the source of the outbreak suggests that *Salmonella* is contaminating alfalfa seed in the farm fields or storage facilities. In 1999, the National Advisory Committee on Microbiological Criteria for Foods released recommendations to the sprouting industry to reduce the risk of contamination of alfalfa sprouts [111]. Recommendations for seed production suggested that growers should evaluate their sources of irrigation and monitor the presence of animal production facilities that could inadvertently expose alfalfa crops to contaminated manure. In addition, the committee concluded that good seed cleaning, storage and handling practices were necessary during seed handling and sprouting in order to reduce cross-contamination [111].

Since almost all sprout outbreaks lead back to contaminated seed lots, research has centered on the ability of *S. enterica* to survive on seeds and resist disinfection. Successful decontamination must inactivate *Salmonella* but preserve seed viability. In a study designed to evaluate various chemical treatments for their effectiveness in killing *Salmonella* on alfalfa seeds, contaminated seeds were immersed in solutions containing 20,000 ppm chlorine, 5% trisodium phosphate, 8% hydrogen peroxide, 1% calcium hydroxide, 1% calcinated calcium, 5% lactic acid or 5% citric acid for ten minutes [112]. Several treatments caused reductions of *Salmonella* populations of up to 10^3^ CFU/g when analyzed by direct plating; however, no treatment was able to eliminate the pathogen [112]. During a 1999 *Salmonella* ser. Muenchen outbreak investigation, an implicated sprout grower signed an affidavit stating that seeds were disinfected with a 20,000-ppm chlorine solution for 15 minutes prior to germination. Despite this FDA-recommended disinfection step, there were at least 157 cases of *Salmonella* gastroenteritis resulting from eating these sprouts [80]. One possible explanation is that *Salmonella* may be protected from lethal concentrations of chlorine by lodging within the rough features on the seed surface [113]. 

The dynamics of *Salmonella* replication and growth during the commercial sprouting process are not clearly understood. In an attempt to understand how *Salmonella* survives and grows throughout the sprouting process, Jacquette *et al*. [114] inoculated alfalfa seeds with a *Salmonella* ser. Stanley strain that was isolated from the 1995 sprout outbreak. After a period of soaking, germination, sprouting and refrigeration, *Salmonella* levels increased from ~10^3^ CFU/g to 10^7^ CFU/g. Disinfection procedures were able to reduce CFU numbers, but elimination could not be reliably achieved [114]. This study proved that *S. enterica* can multiply to high cell numbers on alfalfa seeds despite standard disinfection and handling precautions. In another study, *Salmonella* ser. Eimsbuettel and ser. Poona inoculated on alfalfa seeds increased by 3–4 logs during sprouting [115]. Dong *et al*. [116] found that three *S. enterica* strains isolated from previous alfalfa sprout outbreaks were able to survive on the seed surface and internalize into alfalfa sprouts during the germination process. It is possible that the attachment, resistance and survival of *Salmonella* on alfalfa sprouts may be due in part to the rdar morphotype. Barak *et al*. [117] created a transposon mutagenesis library in a *Salmonella* ser. Newport strain isolated from an outbreak associated with contaminated alfalfa seeds. The transposon library was screened to find mutants defective in attachment to alfalfa sprouts. Loss of expression of genes involving the rdar morphotype (*i.e.*, curli and cellulose production) caused a reduction in binding of the serovar Newport isolate to alfalfa sprouts [117]

In summary, alfalfa-related *Salmonella* outbreaks are primarily caused by NTS serovars that are present on contaminated seeds. These strains can survive on alfalfa seeds for protracted periods of time and resist chemical stresses. During the sprouting process, *S. enterica* can internalize into sprouts and multiply to reach high cell numbers.

#### 2.2.3. *Salmonella* Outbreaks Associated with Processed Foods

Over the last 20 years, the food vehicle of many *Salmonella* gastroenteritis outbreaks has been low-moisture foods such as dry cereal, peanut butter, spray-dried milk, infant formula, nuts and dry seasonings. The low water activity of these foods does not typically support the growth of pathogens. However, *Salmonella* can survive for long periods of time in low-moisture products, and ingestion of fewer than 10^3^ *S. enterica* cells can still lead to illness [92]. Investigations of outbreaks related to low-moisture foods illustrate themes of *Salmonella*’s ability to persist in the food-processing environment. For a comprehensive review of *Salmonella* survival in low-moisture foods, see [118]. For the purpose of this review, we wish only to highlight outbreaks where *S. enterica* strains are exceptional in their ability to cross-contaminate, resist, and survive in this environment, as well as the foods themselves.

One of the pre-requisites for *Salmonella* to persist in food-processing environments and cause cross-contamination is the ability of cells to attach to surfaces and survive there. Chia *et al*. [119] found that *S. enterica* strains isolated from food-processing environments could attach to a variety of surfaces that are commonly used in food processing, such as stainless steel, teflon, glass, rubber and polyurethane. Because *Salmonella* can attach to all of these surfaces, cells can easily be transferred from one surface to another. A study looking at cross-contamination of surfaces in oil meal plants found *S. enterica* isolates on the processing floor, in dust, on the gloves and boots of operators and on tools [120]. To obtain data on cross-contamination, investigators disinfected the boots of plant workers before they began their normal operations for the day. Within one day of disinfection, all workers’ boots tested 100% positive for *Salmonella*. Ultimately, investigators had to recommend restricting the movement of workers from one area of the building to others [120]. In 1998, an outbreak of 209 reported cases of Salmonellosis was associated with ingestion of toasted oat cereal [88]. FDA officials tested potential areas of cross-contamination and found low levels of *Salmonella* throughout the entire processing plant. It was concluded that equipment, air-handling systems and traffic flow had cross-contaminated the plant [88]. 

Cross-contamination by *S. enterica* serovars is not limited to highly processed foods. *Salmonella* contamination in the poultry industry (not reviewed here) is also a well-known problem. Marin *et al*. [121] identified the potential risk factors for *Salmonella* contamination in 44 broiler and 51 layer farms and determined the biofilm-forming capacity of the strains that were isolated. 41.3% of broiler houses tested were contaminated with *Salmonella,* and approximately 50% of strains isolated were able to produce a biofilm. The most important risk factors for *Salmonella* contamination were determined to be dust, environmental surfaces (*i.e.*, wall crevices, floor joints) and chicken feces. Rodents, flies and beetles also played an important role in the recirculation of *Salmonella* in laying hen houses because they were able to taint the feed and house surfaces. Finally, the use of glutaraldehyde (50% vol/vol), formaldehyde (37% vol/vol) and hydrogen peroxygen (35% vol/vol) at a concentration of 1.0% in field conditions were found to be inadequate for *Salmonella* elimination irrespective of the serotype, the biofilm development capacity and the disinfectant contact time [121].

Since *Salmonella* spp. are known to form the rdar morphotype under low-moisture conditions and it confers on *Salmonella* the ability to attach, resist and survive, it may be an important adaptation for *Salmonella* in the factory environment. A correlation between biofilm capacity and persistence in factory environments has been reported. One-hundred eleven strains of serovars Agona, Montevideo, Senftenberg and Typhimurium were isolated from feed and fish meal factories, and several of these strains had persisted for at least three years [122]. Vestby *et al*. [123] hypothesized that several of these clones would be relatively strong biofilm producers because of their persistence in factories. When comparing biofilm formation capacity to serovar Typhimurium, which is rarely isolated from factories but known to be endemic in local avian wildlife [124], serovars Agona and Montevideo produced 423% and 390% more biofilm [123]. 

In many studies, *Salmonella* strains isolated from the environment have been found to produce biofilms. Solomon *et al*. [125] analyzed a collection of 71 strains isolated from clinical, produce and meat sources. Curli fimbriae were produced by 100% of clinical and meat isolates, and 80% of produce isolates. Cellulose was expressed in clinical (73%), meat (84%) and produce (52%) isolates. In another study, a total of 122 *Salmonella* strains were isolated from humans, animals or food, and all strains were found to produce biofilm [126]. Patel and Sharma [127] tested biofilm formation from five *S. enterica* isolates that were associated with produce outbreaks: serovar Thompson 2051H; Tennessee 2053N; Negev 26 H; Braenderup; and Newport. All formed biofilms, with ser. Tennessee and ser. Thompson forming the greatest amount.

In addition to biofilm formation, *S. enterica *isolates have the ability to resist desiccation and heat stress. These traits are well-demonstrated in studies of *Salmonella* outbreaks resulting from contaminated chocolate. Outbreaks associated with chocolate first appeared in the 1970s; a *Salmonella* ser. Durham epidemic linked to contaminated cocoa caused infections in 110 people [91]. A few years later, an outbreak occurred in North America involving chocolate containing *Salmonella* ser. Eastbourne; 217 people were infected [92,93]. An outbreak in the UK in 1982 that infected 245 people was traced to chocolate bars contaminated with *Salmonella* ser. Nepoli [94]. In Norway, more than 300 people were affected after consuming chocolate contaminated with *Salmonella* ser. Typhimurium [96]. In many of these outbreaks, cocoa beans or cocoa powder were suspected to be contaminated with *Salmonella* prior to their use in chocolate production. Under these conditions, *Salmonella* must survive desiccation, heat treatments during chocolate processing and survive in chocolate on store shelves for long periods of time. Most bacteria capable of causing foodborne illness do not grow below a water activity of 0.85 [128], however, *Salmonella* can survive in chocolate, which has a water activity of 0.4–0.5 [129]. *S. enterica* cells surviving in low-water activity foods are more tolerant to heat processing. During processing, chocolate is heated to 70-80°C for 8-24 hours but *Salmonella* is not destroyed [130]. *Salmonella* also can survive for long periods of time in processed chocolate products. Tamminga *et al*. [129] reported recovery of *S. enterica* from chocolate after nine months of storage. In a later study, these authors were able to recover *Salmonella* from chocolate after 19 months of storage [131].

The number of NTS cells needed to cause gastroenteritis is generally high [132], but in the case of chocolate-related outbreaks, low cell numbers can cause infection. An average of 2.5 *Salmonella* ser. Eastbourne organisms per gram of chocolate was found in infected person’s homes in the 1973 outbreak [92]. Investigators concluded that no more than 1000 cells could have caused the infection, which was the number estimated to be in a one pound bag of chocolate. However, the authors stated that technical difficulties in the recovery of *Salmonella* from chocolate might underestimate the actual number of cells per gram of chocolate. In the *Salmonella* ser. Nima outbreak of 1989 (Table 1), concentrations as low as 0.043 cells per gram were found in chocolate [95]. In the most recent *Salmonella* ser. Oranienburg outbreak, there were an estimated –one to three cells per gram of chocolate [97]. Although the number of *S. enterica* cells per gram of chocolate is low, the matrix of chocolate may protect *Salmonella* from the acidic conditions of the stomach, thereby increasing the levels of viable cells that reach the intestine [133].

Tomatoes, sprouts, and chocolate are just three examples from the myriad of food products and sources that have been implicated in outbreaks of Salmonella gastroenteritis. These examples illustrate the diverse adaptations of *S. enterica* subsp. *enterica* for persistence and survival, as well as the inherent difficulty in eradication—all factors that contribute to human infection.

### 2.3. Genomic—and Population—based Studies of Salmonella and Related Enteric Pathogens

Based on the multitude of serovar types that have been implicated in *Salmonella* outbreaks and sporadic infections, there is seemingly a large diversity of *S. enterica* isolates. However, on the basis of sequence identity, we know that most serovars are closely related. As stated above, *S. enterica* subsp. *enterica* serovars can be loosely grouped as host-generalist, with the ability to colonize and infect multiple animal species (*i.e.*, Typhimurium, Enteritidis, Heidelberg), host-adapted, such as Choleraesuis in swine and Dublin in cattle, and then host-restricted, such as serovars Typhi and Paratyphi in humans and Gallinarum in poultry. With the advent of genome sequencing and genome-based microarrays 10–15 years ago, it was assumed that the host-specificities of *S. enterica* serovars would be explained by the presence or absence of specific genes [134]. However, the distinction between serovars has turned out to be more complicated than previously thought. 

Several genomic-based studies have begun to reveal more information about the population structure of *S. enterica* subsp. *enterica*. Using resequencing array technology, Didelot *et al*. [135] demonstrated that there are at least five different lineages within *S. enterica*; these lineages have also been classified as part of two larger phylogenetic clades [136]. Recombination between isolates of the same lineage was significantly greater than between lineages, suggesting that barriers between lineages might exist, possibly due to physical separation as a consequence of host adaptation or to sequence divergence [135]. Serovars Enteritidis and Typhimurium were the central members for two separate lineages and appeared to be monophyletic in origin. This suggests that most Enteritidis and Typhimurium isolates are clonal, with the main differences between isolates due to mutations. *Salmonella* ser. Typhimurium ST313 is a notable exception to this rule. Other serovars, such as Newport and Paratyphi B are polyphyletic in origin, consisting of several distinct groupings within the serovar [137]. Isolates within these serovars have evidence of extensive recombination and often appear to have different origins, despite sharing the surface antigens that were detected by serotyping. Most *S. enterica* serovars are assumed to lie somewhere between these polyphyletic groups and the most highly clonal group, which is *Salmonella* ser. Typhi [138]. 

In general, the evolution of *S. enterica* serovars towards becoming host-adapted, and ultimately host-restricted, is characterized by an accumulation of pseudogenes (loss of gene function) [139], as opposed to physical loss of genes from the chromosome. Recently, there have been efforts to identify pseudogenes that are common between host-adapted and host-restricted serovars. Betancor *et al*. [14] compared the genomes of four serovar Dublin isolates with 29 serovar Enteritidis isolates and matched the identified pseudogenes to the published serovar Gallinarum sequence (strain 287/91). As expected, Dublin and Gallinarum each had approximately three times more pseudogenes than Enteritidis; however, only 21 pseudogenes common to Dublin and Gallinarum represented active genes in Enteritidis. Nine of these pseudogenes were also present in serovar Choleraesuis, and two were in common with serovar Typhi and Paratyphi A isolates. One of the common pseudogenes, *shdA*, has been implicated in intestinal persistence and fecal shedding of *Salmonella* ser. Typhimurium in the mouse model of infection [140,141]. One of the hallmarks of host-adaptation or -restriction is the gain of a systemic mode of infection, presumably at the expense of intestinal persistence [142]. Further research into the role of pseudogenes in host adaptation should yield valuable information about the *S. enterica* lifecycle.

The recent explosion of genome sequencing of different *S. enterica* serovars [137,142,143,144,145,146,147] is moving towards defining a core genome of *S. enterica* subsp. *enterica* [148,149]. From the 73 subsp. *enterica* genomes that were available at the time of analysis, the core genome was determined to consist of 2882 genes [149]; the order of genes and their sequence was highly conserved within the core genome. Most *S. enterica* subsp. *enterica* isolates also have hotspots of unique genes, which occur in relatively similar positions within the genome. From 73 subsp. *enterica* genomes analyzed, over 7000 unique genes were identified [149]. The majority of differences between serovars were due to the presence or absence of bacteriophage, plasmids or other mobile elements [143,144], fimbrial operons [24,136] and the loss of metabolic functions [136,144]. Analysis of the unique or highly variable genes within subsp. *enterica* did not yield any obvious phylogenetic information [149] but was informative when analyzed on a lineage-by-lineage basis [136,144]. The observed variation resulting from recombination and mutation has enabled *Salmonella* to be remarkably adaptable, expanding to fill a spectrum of new niches and responding to environmental challenges. 

The genomic variation between different groups or lineages of *S. enterica* subsp. *enterica* could mean that the ecology, lifecycle and transmission characteristics are also different [136]. For example, serovars Typhi and Paratyphi A have lost the function of several genes relating to intestinal persistence and pathogenesis [139], in addition to numerous fimbrial operons that are used for attachment to host cells [24]. It is hypothesized that Typhi and Paratyphi A are similar due to convergent evolution that has occurred under selection pressure within the human host [150]. It is well known that *Salmonella* ser. Typhi has unique factors relating to host persistence, generating a carrier state, possibly through the colonization of gallstones [15]. It has also been speculated that because of a restricted host range, a long-term reservoir would be necessary for survival of serovar Typhi [1]. Genomic analysis of 19 Typhi isolates showed that evolution was dominated by genetic drift, rather than recombination or gene acquisition, which indicated that carriers were the primary sources of typhoid infections [138]. Transmission of serovar Typhi, then, would be expected to occur primarily through human-human contact and the fecal-oral route, and this is what has been observed [151]. Surprisingly, analysis of the host-restricted serovar Gallinarum, which causes avian typhoid, showed that Gallinarum and Typhi had many of the same patterns of gene loss [142]. This suggests that host adaptation within *S. enterica* subsp. *enterica* involves loss of the intestinal lifestyle, coupled with an ability to cause systemic infection. This niche specialization may also reduce the ability of host-adapted serovars to survive in the external environment [136,142].

There is increasing correlation between curli expression, formation of the rdar morphotype and a host-generalist lifestyle. For the past 10-20 years, *Salmonella* serovars Typhimurium and Enteritidis have been the most common causes of human gastroenteritis worldwide [8]. As well as being able to infect many animal hosts [8], these serovars have retained respiration under anaerobic conditions, as well as a broad substrate spectrum for metabolism [144]; presumably, this allows them to have a flexible niche space. Solano *et al*. [42] found that 93% of ~200 natural serovar Enteritidis isolates were rdar-positive. Romling *et al*. [152] analyzed ~800 Enteritidis and Typhimurium isolates from patients, food and animals, and over 90% of isolates were rdar-positive. Interestingly, all rdar-negative Typhimurium isolates were members of var. Copenhagen, which causes an invasive disease in pigeons. Isolates from host-adapted or -restricted serovars Choleraesuis, Gallinarum and Typhi were all rdar-negative, with the exception of one Gallinarum isolate, strongly suggesting that host-adaptation was associated with loss of the rdar morphotype [152]. Solomon *et al*. [125] and our group analyzed a wider diversity of isolates in subsp. *enterica *[43], as well as other *S. enterica* subspecies, and *S. bongori* as part of the SARC collection [153] and showed that the rdar morphotype was widely conserved throughout the *Salmonella* genus. As expected, we found that serovars Choleraesuis and Paratyphi (A, B or C) were almost entirely rdar-negative. For serovar Typhi, the first two isolates we tested were erroneously reported to be rdar-positive [43]; however, in further testing, no rdar-positive isolates were identified from >100 Typhi isolates analyzed (AP White, SL Stocki and KE Sanderson, unpublished). Both curli operons (*csgBAC* and *csgDEFG)* are present in serovar Typhi, so it is assumed that the rdar morphotype is shut off via regulatory mutations. We have also reported that *S. enterica* subsp. *arizonae* isolates were negative for the rdar morphotype [153]. Not as much is known about subsp. *arizonae*, but it is possible that these isolates are host-adapted to reptiles [154]. *S. enterica* subsp. *arizonae* isolates are known to have pseudogenes in the curli operons [24], as well as serovar Paratyphi A [139]. 

Recent studies in *E. coli* show a similar trend between a host-generalist lifestyle and conservation of the rdar morphotype. We analyzed 284 human, livestock and environmental *E. coli* isolates and generated a phylogenetic tree based on comparisons of three conserved intergenic regions [3]. Isolates that were nearly identical to each other (phylogroup B1), and thus predicted to be host-generalist, were ~90% rdar-positive, whereas isolates with longer branch lengths (phylogroup B2 and part of group D) that were predicted to be host-adapted were only ~30% rdar-positive [3]. Meric *et al*. [155] performed a similar comparative analysis of *E. coli* isolates that were obtained from plants or from animal species. The plant-adapted isolates (primarily phylogroup B1) had a significantly higher prevalence of rdar morphotype formation as compared to the animal isolates (primarily group B2). In addition, similar to *Salmonella*, *E. coli* isolates that cause invasive disease (*i.e.*, enteroinvasive *E. coli* and *Shigella*) have lost the ability to form the rdar morphotype [156]. Since the operons for curli and cellulose production were likely present in the common ancestor of *Salmonella* and *E. coli* [25,135,157], it makes sense that trends relating to rdar morphotype expression may have evolved similarly. Nevertheless, despite these observed trends, correlation does not necessarily equal causation, and there is no published evidence that the rdar morphotype is involved in transmission. 

One of the main problems for understanding NTS transmission is that there are no good models to test the relative importance or function of *Salmonella* genes in the transmission process. A chronic infection model was recently developed for serovar Typhimurium in mice designed to mimic the human carrier state of serovar Typhi [158]. Although it is difficult to extrapolate from mice to humans, use of this model will undoubtedly lead to the identification of factors that aid in *Salmonella* ser. Typhi persistence and possible transmission from the carrier state. Many research groups around the world are designing genome-wide screens to study the infectious process for both NTS and typhoid. It is important to consider that there will always be a significant percentage of *Salmonella* genes whose functions are not involved in infection, but rather, are needed for survival in external environments. These genes will be missed in standard infection screening. Most new *S. enterica* subsp. *enterica* genomes still have 20–30% of genes with unknown function. For example, 76% of genes identified by Fricke *et al*. [144] that were unique to a given serovar were annotated as hypothetical proteins, as compared to only 10% of the genes that were absent. Although it is difficult to study NTS transmission, there has been a murine model recently proposed for serovar Typhimurium [159]. In addition to expanding on current transmission models, there is also a need to establish what the real-world conditions are like for human transmission. 

## 3. Commentary: Infectious NTS Isolates in Africa

There is a prevailing thought in North America that NTS isolates are not life-threatening pathogens, simply causing a self-limiting gastroenteritis that usually does not require hospitalization. In Africa, however, NTS isolates cause an invasive type of disease that has a mortality rate as high as 25%, especially in young children or HIV-positive individuals [10,160]. These infectious NTS (iNTS) isolates represent a new variant group of serovar Typhimurium (ST313) that have many of the hallmarks of host-adaptation, with accumulation of pseudogenes in pathways that are also inactivated in serovars Typhi or Paratyphi A [9]. Genetic characterization of the iNTS pathogens helps in the design of effective treatment strategies [146]. However, these authors have stated that the largest gap in knowledge has to do with transmission. While the public health situation in the industrialized world cannot be compared to Africa, we feel that there is also a large gap in understanding the transmission process for more “typical” NTS isolates. 

## 4. Conclusions

In writing this review, we felt that for the reader to gain a better perspective on *Salmonella* transmission, we needed to describe laboratory models of *Salmonella* survival in addition to presenting real-world examples of *S. enterica* outbreaks. It is apparent that even though the vehicles for transmission are often well-defined, there is still much to learn about where and how *Salmonella* persists. *Salmonella* survival adaptations like the rdar morphotype have been studied in the laboratory, but it is unknown how relevant these adaptations are to the persistence of *Salmonella* in a real outbreak. After reviewing outbreaks in several food vehicles, a pattern emerged whereby persistence of *Salmonella* in the food processing environment was due to increased resistance, ability to cross-contaminate, and long-term survival, characteristics that have been ascribed to the rdar morphotype, It is increasingly clear that the form of *Salmonella* in the environment is different than *Salmonella* in the host. To intervene effectively in industrial processes and ensure safe food handling as we move into the future, we need to understand the strategies *S. enterica* has evolved that enable it to transmit so efficiently. This is increasingly relevant with the globalization of our food supply and the use of centralized processing and storage facilities. In our view, more research should be aimed at identifying the genes involved in the transmission *S. enterica*, which requires that better transmission models be developed. In addition, there is a continued need for increased epidemiological surveillance to identify reservoirs in the environment. *Salmonella* has adapted remarkably well to diverse environments and there will not be a simple solution for reducing the prevalence of *Salmonella* infections. However, increasing our knowledge about transmission can only help to minimize its worldwide impact.

## Supplementary

### Graphical Representation of Waldner *et al.* Manuscript

An overview of the most common *S. enterica* subsp. *enterica* serovars, connected to each other in terms of host-adaptation, with disease and transmission characteristics listed below. Host-generalist serovars are associated with many different food sources, with tomatoes, alfalfa sprouts and chocolate being discussed in this review. Host-specific serovars listed are either human-specific (Typhi, Paratyphi A,B,C) or fowl-specific (Gallinarum). *Salmonella* ser. Typhimurium var. Copenhagen is listed towards host-specific because it is associated with an invasive, typhoid-like disease in birds and is negative for the rdar morphotype. The infectious NTS (iNTS) isolates causing infections in Africa with a high mortality rate appear to have many of the hallmarks of host-adaptation, but their other transmission characteristics have not yet been determined; this is why the question mark is listed. During the evolutionary process toward host-adaptation and host-specificity, serovars have often accumulated pseudogenes in *shdA* and other genes associated with intestinal persistence. There is a high prevalence of rdar morphotype (>80%) in host-generalist serovars, whereas isolates of host-adapted or -specific serovars are almost entirely negative. The rdar morphotype has been linked to long-term survival in non-host environments, which fits with the proposed transmission profiles of the serovars listed.

## References

[1] Blaser M.J., Kirschner D. (2007). The equilibria that allow bacterial persistence in human hosts. Nature.

[2] Savageau M.A. (1983). *Escherichia coli* habitats, cell types, and molecular mechanisms of gene control. Am. Nat..

[3] White A.P., Sibley K.A., Sibley C.D., Wasmuth J.D., Schaefer R., Surette M.G., Edge T.A., Neumann N.F. (2011). Intergenic sequence comparison of *Escherichia coli* isolates reveals lifestyle adaptations but not host specificity. Appl. Environ. Microbiol..

[4] Santamaria J., Toranzos G.A. (2003). Enteric pathogens and soil: A short review. Int. Microbiol..

[5] Le Minor L., Popoff M.Y. (1987). Designation of *Salmonella enterica* sp. Nov., nom. Rev., as the type and only species of the genus *Salmonella*: Request for an opinion. Int. J. Syst. Bacteriol..

[6] Reeves M.W., Evins G.M., Heiba A.A., Plikaytis B.D., Farmer Iii J.J. (1989). Clonal nature of *Salmonella typhi* and its genetic relatdeness to other salmonellae as shown by multilocus enzyme electrophoresis, and proposal of *Salmonella bongori* comb. Nov. J. Clin. Microbiol..

[7] Grimont P.A.D., Weill F.X. (2007). Antigenic formulae of the Salmonella serovars.

[8] Callaway T.R., Edrington T.S., Anderson R.C., Byrd J.A., Nisbet D.J. (2007). Gastrointestinal microbial ecology and the safety of our food supply as related to *Salmonella*. J. Anim. Sci..

[9] Kingsley R.A., Msefula C.L., Thomson N.R., Kariuki S., Holt K.E., Gordon M.A., Harris D., Clarke L., Whitehead S., Sangal V. (2009). Epidemic multiple drug resistant *Salmonella* Typhimurium causing invasive disease in sub-saharan Africa have a distinct genotype. Genome Res..

[10] Feasey N.A., Dougan G., Kingsley R.A., Heyderman R.S., Gordon M.A. (2012). Invasive nontyphoidal *Salmonella* disease: An emerging and neglected tropical disease in Africa. Lancet.

[11] Majowicz S.E., Musto J., Scallan E., Angulo F.J., Kirk M., O'Brien S.J., Jones T.F., Fazil A., Hoekstra R.M. (2010). The global burden of nontyphoidal *Salmonella* gastroenteritis. Clin. Infect. Dis..

[12] Crump J.A., Mintz E.D. (2010). Global trends in typhoid and paratyphoid fever. Clin. Infect. Dis..

[13] Voetsch A.C., Van Gilder T.J., Angulo F.J., Farley M.M., Shallow S., Marcus R., Cieslak P.R., Deneen V.C., Tauxe R.V. (2004). Foodnet estimate of the burden of illness caused by nontyphoidal *Salmonella* infections in the united states. Clin. Infect. Dis..

[14] Betancor L., Yim L., Martinez A., Fookes M., Sasias S., Schelotto F., Thomson N., Maskell D., Chabalgoity J.A. (2012). Genomic comparison of the closely related *Salmonella enterica* serovars Enteritidis and Dublin. Open Microbiol. J..

[15] Gonzalez-Escobedo G., Marshall J.M., Gunn J.S. (2011). Chronic and acute infection of the gall bladder by *Salmonella* Typhi: Understanding the carrier state. Nat. Rev. Microbiol..

[16] Levine M.M., Black R.E., Lanata C. (1982). Precise estimation of the numbers of chronic carriers of *Salmonella typhi* in Santiago, Chile, an endemic area. J. Infect. Dis..

[17] Stokes A., Clarke C. (1916). A search for typhoid carriers among 800 convalescents. Lancet.

[18] Vogelsang T.M., Boe J. (1948). Temporary and chronic carriers of *Salmonella typhi* and *Salmonella paratyphi* B. J. Hyg..

[19] Buchwald D.S., Blaser M.J. (1984). A review of human salmonellosis: Ii. Duration of excretion following infection with nontyphi *Salmonella*. Rev. Infect. Dis..

[20] Winfield M.D., Groisman E.A. (2003). Role of nonhost environments in the lifestyles of *Salmonella* and *Escherichia coli*. Appl. Environ. Microbiol..

[21] Spector M.P., Kenyon W.J. (2012). Resistance and survival strategies of *Salmonella enterica* to environmental stresses. Food Res. Int..

[22] Davies R.H., Breslin M. (2003). Persistence of *Salmonella enteritidis* phage type 4 in the environment and arthropod vectors on an empty free-range chicken farm. Environ. Microbiol..

[23] Snow L.C., Davies R.H., Christiansen K.H., Carrique-Mas J.J., Cook A.J., Evans S.J. (2010). Investigation of risk factors for *Salmonella* on commercial egg-laying farms in Great Britain, 2004-2005. Vet. Rec..

[24] Nuccio S.P., Thomson N.R., Fookes M.C., Baumler A.J. (2011). Fimbrial signature arrangements in salmonella. Salmonella: From genome to function.

[25] Baumler A.J., Gilde A.J., Tsolis R.M., van der Velden A.W., Ahmer B.M., Heffron F. (1997). Contribution of horizontal gene transfer and deletion events to development of distinctive patterns of fimbrial operons during evolution of *Salmonella* serotypes. J. Bacteriol..

[26] Doran J.L., Collinson S.K., Burian J., Sarlos G., Todd E.C., Munro C.K., Kay C.M., Banser P.A., Peterkin P.I., Kay W.W. (1993). DNA-based diagnostic tests for *Salmonella* species targeting *agfA*, the structural gene for thin, aggregative fimbriae. J. Clin. Microbiol..

[27] Collinson S.K., Emody L., Muller K.H., Trust T.J., Kay W.W. (1991). Purification and characterization of thin, aggregative fimbriae from *Salmonella enteritidis*. J. Bacteriol..

[28] Collinson S.K., Doig P.C., Doran J.L., Clouthier S., Trust T.J., Kay W.W. (1993). Thin, aggregative fimbriae mediate binding of *Salmonella enteritidis* to fibronectin. J. Bacteriol..

[29] Collinson S.K., Emody L., Trust T.J., Kay W.W. (1992). Thin aggregative fimbriae from diarrheagenic *Escherichia coli*. J. Bacteriol..

[30] Romling U., Sierralta W.D., Eriksson K., Normark S. (1998). Multicellular and aggregative behaviour of *Salmonella typhimurium* strains is controlled by mutations in the *agfD* promoter. Mol. Microbiol..

[31] Romling U., Bian Z., Hammar M., Sierralta W.D., Normark S. (1998). Curli fibers are highly conserved between *Salmonella typhimurium* and *Escherichia coli* with respect to operon structure and regulation. J. Bacteriol..

[32] White A.P., Gibson D.L., Collinson S.K., Banser P.A., Kay W.W. (2003). Extracellular polysaccharides associated with thin aggregative fimbriae of *Salmonella enterica* serovar Enteritidis. J. Bacteriol..

[33] Romling U., Rohde M., Olsen A., Normark S., Reinkoster J. (2000). AgfD, the checkpoint of multicellular and aggregative behaviour in *Salmonella typhimurium* regulates at least two independent pathways. Mol. Microbiol..

[34] Gibson D.L., White A.P., Snyder S.D., Martin S., Heiss C., Azadi P., Surette M., Kay W.W. (2006). *Salmonella* produces an o-antigen capsule regulated by AgfD and important for environmental persistence. J. Bacteriol..

[35] de Rezende C.E., Anriany Y., Carr L.E., Joseph S.W., Weiner R.M. (2005). Capsular polysaccharide surrounds smooth and rugose types of *Salmonella enterica* serovar Typhimurium DT104. Appl. Environ. Microbiol..

[36] Latasa C., Roux A., Toledo-Arana A., Ghigo J., Gamazo C., Penades J.R., Lasa I. (2005). Bapa, a large secreted protein required for biofilm formation and host colonization of *Salmonella enterica* serovar Enteritidis. Mol. Microbiol..

[37] Chapman M.R., Robinson L.S., Pinkner J.S., Roth R., Heuser J., Hammar M., Normark S., Hultgren S.J. (2002). Role of *Escherichia coli* curli operons in directing amyloid fiber formation. Science.

[38] Collinson S.K., Parker J.M., Hodges R.S., Kay W.W. (1999). Structural predictions of AgfA, the insoluble fimbrial subunit of *Salmonella* thin aggregative fimbriae. J. Mol. Biol..

[39] Larsen P., Nielsen J.L., Dueholm M.S., Wetzel R., Otzen D., Nielsen P.H.r. (2007). Amyloid adhesins are abundant in natural biofilms. Environ. Microbiol..

[40] Anriany Y.A., Weiner R.M., Johnson J.A., De Rezende C.E., Joseph S.W. (2001). *Salmonella enterica* serovar Typhimurium DT104 displays a rugose phenotype. Appl. Environ. Microbiol..

[41] Scher K., Romling U., Yaron S. (2005). Effect of heat, acidification, and chlorination on *Salmonella enterica* serovar Typhimurium cells in a biofilm formed at the air-liquid interface. Appl. Environ. Microbiol..

[42] Solano C., Garcia B., Valle J., Berasain C., Ghigo J.M., Gamazo C., Lasa I. (2002). Genetic analysis of *Salmonella enteritidis* biofilm formation: Critical role of cellulose. Mol. Microbiol..

[43] White A.P., Gibson D.L., Kim W., Kay W.W., Surette M.G. (2006). Thin aggregative fimbriae and cellulose enhance long-term survival and persistence of *Salmonella*. J. Bacteriol..

[44] Stocki S.L., Annett C.B., Sibley C.D., McLaws M., Checkley S.L., Singh N., Surette M.G., White A.P. (2007). Persistence of *Salmonella* on egg conveyor belts is dependent on the belt type but not on the rdar morphotype. Poult. Sci..

[45] Uhlich G.A., Cooke P.H., Solomon E.B. (2006). Analyses of the red-dry-rough phenotype of an *Escherichia coli* O157:H7 strain and its role in biofilm formation and resistance to antibacterial agents. Appl. Environ. Microbiol..

[46] Austin J.W., Sanders G., Kay W.W., Collinson S.K. (1998). Thin aggregative fimbriae enhance *Salmonella enteritidis* biofilm formation. FEMS Microbiol. Lett..

[47] Ryu J.H., Beuchat L.R. (2005). Biofilm formation by *Escherichia coli* O157:H7 on stainless steel: Effect of exopolysaccharide and curli production on its resistance to chlorine. Appl. Environ. Microbiol..

[48] Apel D., White A.P., Grassl G.A., Finlay B.B., Surette M.G. (2009). Long-term survival of *Salmonella enterica* serovar Typhmurium reveals an *Infect.* state that is underrepresented on laboratory media containing bile salts. Appl. Environ. Microbiol..

[49] White A.P., Gibson D.L., Grassl G.A., Kay W.W., Finlay B.B., Vallance B.A., Surette M.G. (2008). Aggregation via the red, dry, and rough morphotype is not a virulence adaptation in *Salmonella enterica* serovar Typhimurium. Infect. Immun..

[50] Knudsen G.M., Nielsen M.-B., Grassby T., Danino-Appleton V., Thomsen L.E., Colquhoun I.J., Brocklehurst T.F., Olsen J.E., Hinton J.C.D. (2012). A third mode of surface-associated growth: Immobilization of *Salmonella enterica* serovar Typhimurium modulates the RpoS-directed transcriptional programme. Environ.Microbiol.

[51] White A.P., Weljie A.M., Apel D., Zhang P., Shaykhutdinov R., Vogel H.J., Surette M.G. (2010). A global metabolic shift is linked to *Salmonella* multicellular development. PLoS One.

[52] Grantcharova N., Peters V., Monteiro C., Zakikhany K., Romling U. (2010). Bistable expression of *csgD* in biofilm development of *Salmonella enterica* serovar Typhimurium. J. Bacteriol..

[53] Siebring J., Sorg R.A., Herber M., Kuipers O.P., Filloux A.A.M. (2012). Take it or leave it: Mechanisms underlying bacterial bistable regulatory networks. Bacterial Regulatory Networks.

[54] Lewis K. (2010). Persister cells. Annu. Rev. Microbiol..

[55] Gunn J.S. (2000). Mechanisms of bacterial resistance and response to bile. Microbes Infect..

[56] Roszak D.B., Grimes D.J., Colwell R.R. (1984). Viable but nonrecoverable stage of *Salmonella enteritidis* in aquatic systems. Can. J. Microbiol..

[57] Gupte A.R., de Rezende C.L.E., Joseph S.W. (2003). Induction and resuscitation of viable but nonculturable *Salmonella enterica* serovar Typhimurium DT104. Appl. Environ. Microbiol..

[58] Panutdaporn N., Kawamoto K., Asakura H., Makino S.I. (2006). Resuscitation of the viable but non-culturable state of *Salmonella enterica* serovar Oranienburg by recombinant resuscitation-promoting factor derived from *Salmonella* Typhimurium strain LT2. Int. J. Food Microbiol..

[59] Reissbrodt R., Heier H., Tschape H., Kingsley R.A., Williams P.H. (2000). Resuscitation by ferrioxamine e of stressed *Salmonella enterica* serovar Typhimurium from soil and water microcosms. Appl. Environ. Microbiol..

[60] Reissbrodt R., Rienaecker I., Romanova J.M., Freestone P.P.E., Haigh R.D., Lyte M., Tschape H., Williams P.H. (2002). Resuscitation of *Salmonella enterica* serovar Typhimurium and enterohemorrhagic *Escherichia coli* from the viable but nonculturable state by heat-stable enterobacterial autoinducer. Appl. Environ. Microbiol..

[61] Caro A., Got P., Baleux B. (1999). Physiological changes of *Salmonella typhimurium* cells under osmotic and starvation conditions by image analysis. FEMS Microbiol. Lett..

[62] Lesne J., Berthet S., Binard S., Rouxel A., Humbert F. (2000). Changes in culturability and virulence of *Salmonella typhimurium* during long-term starvation under desiccating conditions. Int. J. Food Microbiol..

[63] Smith R.J., Newton A.T., Harwood C.R., Barer M.R. (2002). Active but nonculturable cells of *Salmonella enterica* serovar Typhimurium do not infect or colonize mice. Microbiol..

[64] Kamruzzaman M., Udden S.M.N., Cameron D.E., Calderwood S.B., Nair G.B., Mekalanos J.J., Faruque S.M. (2010). Quorum-regulated biofilms enhance the development of conditionally viable, environmental *Vibrio cholerae*. Proc. Natl. Acad. Sci. USA.

[65] CDC (2011). Vital signs: Incidence and trends of infection with pathogens transmitted commonly through food-foodborne diseases active surveillance network, 10 U.S. Sites, 1996-2010. MMWR.

[66] CDC (2009). Preliminary foodnet data on the incidence of infection with pathogens transmitted commonly through food, 10 states. MMWR.

[67] Hedberg C.W., Angulo F.J., White K.E., Langkop C.W., Schell W.L., Stobierski M.G., Schuchat A., Besser J.M., Dietrich S. (1999). Outbreaks of salmonellosis associated with eating uncooked tomatoes: Implications for public health. Epidemiol. Infect..

[68] Cummings K., Barrett E., Mohle-Boetani J.C., Brooks J.T., Farrar J., Hunt T., Fiore A., Komatsu K., Werner S.B., Slutsker L. (2001). A multistate outbreak of *Salmonella enterica* serotype Baildon associated with domestic raw tomatoes. Emerg. Infect. Dis..

[69] Greene S.K., Daly E.R., Talbot E.A., Demma L.J., Holzbauer S., Patel N.J., Hill T.A., Walderhaug M.O., Hoekstra R.M., Lynch M.F., Painter J.A. (2008). Recurrent multistate outbreak of *Salmonella* newport associated with tomatoes from contaminated fields, 2005. Epidemiol. Infect..

[70] CDC (2005). Outbreaks of *Salmonella* infections associated with eating roma tomatoes-united states and canada, 2004. MMWR.

[71] Gupta S.K., Nalluswami K., Snider C., Perch M., Balasegaram M., Burmeister D., Lockett J., Sandt C., Hoekstra R.M., Montgomery S. (2007). Outbreak of *Salmonella* Braenderup infections associated with roma tomatoes, northeastern United States, 2004: A useful method for subtyping exposures in field investigations. Epidemiol. Infect..

[72] Sandt C.H., Krouse D.A., Cook C.R., Hackman A.L., Chmielecki W.A., Warren N.G. (2006). The key role of pulsed-field gel electrophoresis in investigation of a large multiserotype and multistate food-borne outbreak of *Salmonella* infections centered in Pennsylvania. J. Clin. Microbiol..

[73] CDC (2007). Multistate outbreaks of Salmonella infections associated with raw tomatoes eaten in restaurants--United States, 2005-2006. MMWR.

[74] Behravesh C.B., Blaney D., Medus C., Bidol S.A., Phan Q., Soliva S., Daly E.R., Smith K., Miller B., Taylor T. (2012). Multistate outbreak of *Salmonella* serotype Typhimurium infections associated with consumption of restaurant tomatoes, USA, 2006: Hypothesis generation through case exposures in multiple restaurant clusters. Epidemiol. Infect..

[75] Mahon B.E., Ponka A., Hall W.N., Komatsu K., Dietrich S.E., Siitonen A., Cage G., Hayes P.S., Lambert-Fair M.A., Bean N.H., Griffin P.M., Slutsker L. (1997). An international outbreak of *Salmonella* infections caused by alfalfa sprouts grown from contaminated seeds. J. Infect. Dis..

[76] Van Beneden C.A., Keene W.E., Strang R.A., Werker D.H., King A.S., Mahon B., Hedberg K., Bell A., Kelly M.T., Balan V.K., Mac Kenzie W.R., Fleming D. (1999). Multinational outbreak of *Salmonella enterica* serotype Newport infections due to contaminated alfalfa sprouts. J. Am. Med. Assoc..

[77] Mohle-Boetani J.C., Farrar J.A., Werner S.B., Minassian D., Bryant R., Abbott S., Slutsker L., Vugia D.J. (2001). *Escherichia coli* O157 and *Salmonella* infections associated with sprouts in California, 1996*-*1998. Ann. Int. Med..

[78] Pezzino G., Miller C., Flahart R., Potsic S.R. (1998). A multi-state outbreak of *Salmonella* serotypes Infantis and Anatum - Kansas and Missouri, 1997. Kansas Medicine: J. Kansas Med. Soc..

[79] Gill C.J., Keene W.E., Mohle-Boetani J.C., Farrar J.A., Waller P.L., Hahn C.G., Cieslak P.R. (2003). Alfalfa seed decontamination in a *Salmonella* outbreak. Emerg. Infect. Dis..

[80] Proctor M.E., Hamacher M., Tortorello M.L., Archer J.R., Davis J.P. (2001). Multistate outbreak of *Salmonella* serovar Muenchen infections associated with alfalfa sprouts grown from seeds pretreated with calcium hypochlorite. J. Clin. Microbiol..

[81] Brooks J.T., Rowe S.Y., Shillam P., Heltzel D.M., Hunter S.B., Slutsker L., Hoekstra R.M., Luby S.P. (2001). *Salmonella* Typhimurium infections transmitted by chlorine-pretreated clover sprout seeds. Am. J. Epidemiol..

[82] Winthrop K.L., Palumbo M.S., Farrar J.A., Mohle-Boetani J.C., Abbott S., Beatty M.E., Inami G., Werner S.B. (2003). Alfalfa sprouts and *Salmonella* Kottbus infection: A multistate outbreak following inadequate seed disinfection with heat and chlorine. J. Food Prot..

[83] CDC (2009). Outbreak of *Salmonella* serotype Saintpaul infections associated with eating alfalfa sprouts - United States, 2009. MMWR.

[84] CDC *Salmonella* newport on alfalfa sprouts. http://www.cdc.gov/salmonella/newport/index.html.

[85] CDC *Salmonella* linked to alfalfa sprouts. http://www.cdc.gov/salmonella/i4512i-/021011/index.html.

[86] CDC *Salmonella* Enteritidis infections on alfalfa sprouts. http://www.cdc.gov/salmonella/sprouts-enteritidis0611/index.html.

[87] Lehmacher A., Bockemuhl J., Aleksic S. (1995). Nationwide outbreak of human salmonellosis in germany due to contaminated paprika and paprika-powdered potato chips. Epidemiol. Infect..

[88] CDC (1998). Multistate outbreak of *Salmonella* serotype Agona infections linked to toasted oats cereal--United States, April-May, 1998. MMWR.

[89] Hiramatsu R., Matsumoto M., Sakae K., Miyazaki Y. (2005). Ability of shiga toxin-producing *Escherichia coli* and *Salmonella* spp. To survive in a desiccation model system and in dry foods. Appl. Environ. Microbiol..

[90] Tsuji H., Hamada K. (1999). Outbreak of salmonellosis caused by ingestion of cuttlefish chips contaminated by both *Salmonella* Chester and *Salmonella* Oranienburg. Jpn. J.Infect. Dis..

[91] Gastrin B., Kampe A., Nystrom K.G., Oden-Johanson B., Wessel G., Zetterberg B. (1972). *Salmonella durham* epidemic caused by contaminated cocoa. Lakartidningen.

[92] Craven P.C., Mackel D.C., Baine W.B., Barker W.H., Gangarosa E.J. (1975). International outbreak of *Salmonella* Eastbourne infection traced to contaminated chocolate. Lancet.

[93] D'Aoust J.Y., Aris B.J., Thisdele P., Durante A., Brisson N., Dragon D., Lachapelle G., Johnston M., Laidley R. (1975). *Salmonella eastbourne* outbreak associated with chocolate. Can. Inst. Food Sci. Technol. J..

[94] Gill O.N., Sockett P.N., Bartlett C.L., Vaile M.S., Rowe B., Gilbert R.J., Dulake C., Murrell H.C., Salmaso S. (1983). Outbreak of *Salmonella napoli* infection caused by contaminated chocolate bars. Lancet.

[95] Hockin J.C., D'Aoust (1989). An international outbreak of *Salmonella nima* from imported chocolate. J. Food Prot..

[96] Kapperud G., Gustavsen S., Hellesnes I., Hansen A.H., Lassen J., Hirn J., Jahkola M., Montenegro M.A., Helmuth R. (1990). Outbreak of *Salmonella typhimurium* infection traced to contaminated chocolate and caused by a strain lacking the 60-megadalton virulence plasmid. J. Clin. Microbiol..

[97] Werber D., Dreesman J., Feil F., van Treeck U., Fell G., Ethelberg S., Hauri A.M., Roggentin P., Prager R., Fisher I.S.T., Behnke S.C., Bartelt E., Weise E., Ellis A., Siitonen A., Andersson Y., Tschape H., Kramer M.H., Ammon A. (2005). International outbreak of *Salmonella* Oranienburg due to German chocolate. BMC Infect. Dis..

[98] Micallef S.A., Rosenberg Goldstein R.E., George A., Kleinfelter L., Boyer M.S., McLaughlin C.R., Estrin A., Ewing L., Jean-Gilles Beaubrun J., Hanes D.E., Kothary M.H., Tall B.D., Razeq J.H., Joseph S.W., Sapkota A.R. (2012). Occurrence and antibiotic resistance of multiple *Salmonella* serotypes recovered from water, sediment and soil on mid-atlantic tomato farms. Environ. Res..

[99] Gaertner J.P., Garres T., Becker J.C., Jimenez M.L., Forstner M.R.J., Hahn D. (2009). Temporal analyses of salmonellae in a headwater spring ecosystem reveals the effects of precipitation and runoff events. J. Water Health.

[100] Haley B.J., Cole D.J., Lipp E.K. (2009). Distribution, diversity, and seasonality of waterborne salmonellae in a rural watershed. Appl. Environ. Microbiol..

[101] Polo F., Figueras M.J., Inza I., Sala J., Fleisher J.M., Guarro J. (1998). Relationship between presence of *Salmonella* and indicators of faecal pollution in aquatic habitats. FEMS Microbiol. Lett..

[102] Polo F., Figueras M.J., Inza I., Sala J., Fleisher J.M., Guarro J. (1999). Prevalence of *Salmonella* serotypes in environmental waters and their relationships with indicator organisms. Antonie Van Leeuwenhoek.

[103] Santo Domingo J.W., Harmon S., Bennett J. (2000). Survival of *Salmonella* species in river water. Curr. Microbiol..

[104] Gaertner J.P., Mendoza J.A., Forstner M.R.J., Hahn D. (2011). Recovery of *Salmonella* from biofilms in a headwater spring ecosystem. J. Water Health.

[105] Hintz L.D., Boyer R.R., Ponder M.A., Williams R.C., Rideout S.L. (2010). Recovery of *Salmonella enterica* newport introduced through irrigation water from tomato (*Lycopersicum esculentum*) fruit, roots, stems, and leaves. HortScience.

[106] Guo X., van Iersel M.W., Chen J., Brackett R.E., Beuchat L.R. (2002). Evidence of association of salmonellae with tomato plants grown hydroponically in inoculated nutrient solution. Appl. Environ. Microbiol..

[107] Miles J.M., Sumner S.S., Boyer R.R., Williams R.C., Latimer J.G., McKinney J.M. (2009). Internalization of *Salmonella enterica* serovar Montevideo into greenhouse tomato plants through contaminated irrigation water or seed stock. J. Food Prot..

[108] Gu G., Hu J., Cevallos-Cevallos J.M., Richardson S.M., Bartz J.A., van Bruggen A.H.C. (2011). Internal colonization of *Salmonella enterica* serovar Typhimurium in tomato plants. PLoS One.

[109] Zhuang R.Y., Beuchat L.R., Angulo F.J. (1995). Fate of *Salmonella montevideo* on and in raw tomatoes as affected by temperature and treatment with chlorine. Appl. Environ.Microbiol..

[110] Backer H.D., Mohle-Boetani J.C., Werner S.B., Abbott S.L., Farrar J., Vugia D.J. (2000). High incidence of extra-intestinal infections in a *Salmonella* Havana outbreak associated with alfalfa sprouts. Pub. Health Rep..

[111] National Advisory Committee on Microbiological Criteria for Food (1999). Microbiological safety evaluations and recommendations on sprouted seeds. Int. J. Food Microbiol..

[112] Weissinger W.R., Beuchat L.R. (2000). Comparison of aqueous chemical treatments to eliminate *Salmonella* on alfalfa seeds. J. Food Prot..

[113] Beuchat L.R., Ryu J.H. (1997). Produce handling and processing practices. Emerg. Infect. Dis..

[114] Jaquette C.B., Beuchat L.R., Mahon B.E. (1996). Efficacy of chlorine and heat treatment in killing *Salmonella stanley* inoculated onto alfalfa seeds and growth and survival of the pathogen during sprouting and storage. Appl. Environ. Microbiol..

[115] Andrews W.H., Mislivec P.B., Wilson C.R., Bruce V.R., Poelma P.L., Gibson R., Trucksess M.W., Young K. (1982). Microbial hazards associated with bean sprouting. J. Assoc. Off. Anal. Chem..

[116] Dong Y., Iniguez A.L., Ahmer B.M.M., Triplett E.W. (2003). Kinetics and strain specificity of rhizosphere and endophytic colonization by enteric bacteria on seedlings of *Medicago sativa* and *Medicago truncatula*. Appl. Environ. Microbiol..

[117] Barak J.D., Gorski L., Naraghi-Arani P., Charkowski A.O. (2005). *Salmonella enterica* virulence genes are required for bacterial attachment to plant tissue. Appl. Environ. Microbiol..

[118] Podolak R., Enache E., Stone W., Black D.G., Elliott P.H. (2010). Sources and risk factors for contamination, survival, persistence, and heat resistance of *Salmonella* in low-moisture foods. J. Food Prot..

[119] Chia T.W.R., Goulter R.M., McMeekin T., Dykes G.A., Fegan N. (2009). Attachment of different *Salmonella* serovars to materials commonly used in a poultry processing plant. Food Microbiol..

[120] Morita T., Kitazawa H., Iida T., Kamata S. (2006). Prevention of *Salmonella* cross-contamination in an oilmeal manufacturing plant. J. Appl. Microbiol..

[121] Marin C., Hernandiz A., Lainez M. (2009). Biofilm development capacity of *Salmonella* strains isolated in poultry risk factors and their resistance against disinfectants. Poult. Sci..

[122] Nesse L.L., Nordby K., Heir E., Bergsjoe B., Vardund T., Nygaard H., Holstad G. (2003). Molecular analyses of *Salmonella enterica* isolates from fish feed factories and fish feed ingredients. Appl. Environ. Microbiol..

[123] Vestby L.K., Moretro T., Langsrud S., Heir E., Nesse L.L. (2009). Biofilm forming abilities of *Salmonella* are correlated with persistence in fish meal- and feed factories. BMC Vet. Res..

[124] Refsum T.r., Handeland K., Baggesen D.L., Holstad G., Kapperud G. (2002). Salmonellae in avian wildlife in norway from 1969 to 2000. Appl. Environ. Microbiol..

[125] Solomon E.B., Niemira B.A., Sapers G.M., Annous B.A. (2005). Biofilm formation, cellulose production, and curli biosynthesis by *Salmonella* originating from produce, animal, and clinical sources. J. Food Prot..

[126] Stepanovic S., Cirkovic I., Mijac V., Svabic-Vlahovic M. (2003). Influence of the incubation temperature, atmosphere and dynamic conditions on biofilm formation by *Salmonella* spp. Food Microbiol..

[127] Patel J., Sharma M. (2010). Differences in attachment of *Salmonella enterica* serovars to cabbage and lettuce leaves. Int. J. Food Microbiol..

[128] Lund B.M., Eklund T. (2000). Control of pH and use of organic acids. Microbiological Safety and Quality of Food.

[129] Tamminga S.K., Beumer R.R., Kampelmacher E.H., van Leusden F.M. (1976). Survival of *Salmonella eastbourne* and *Salmonella typhimurium* in chocolate. J. Hyg..

[130] Goepfert J.M., Biggie R.A. (1968). Heat resistance of *Salmonella typhimurium* and *Salmonella senftenberg* 775W in milk chocolate. Appl. Microbiol..

[131] Tamminga S.K., Beumer R.R., Kampelmacher E.H., van Leusden F.M. (1977). Survival of *Salmonella eastbourne* and *Salmonella typhimurium* in milk chocolate prepared with artificially contaminated milk powder. J. Hyg..

[132] Blaser M.J., Newman L.S. (1982). A review of human salmonellosis: I. Infective dose. Rev. Infect. Dis..

[133] D'Aoust J.Y. (1977). *Salmonella* and the chocolate industry. A review. J. Food Prot..

[134] Chan K., Baker S., Kim C.C., Detweiler C.S., Dougan G., Falkow S. (2003). Genomic comparison of *Salmonella*
*enterica* serovars and *Salmonella*
*bongori* by use of an *S. enterica* serovar Typhimurium DNA microarray. J. Bacteriol..

[135] Didelot X., Bowden R., Street T., Golubchik T., Spencer C., McVean G., Sangal V., Anjum M.F., Achtman M., Falush D., Donnelly P. (2011). Recombination and population structure in *Salmonella enterica*. PLoS Genet..

[136] den Bakker H.C., Switt A.I.M., Govoni G., Cummings C.A., Ranieri M.L., Degoricija L., Hoelzer K., Rodriguez-Rivera L.D., Brown S., Bolchacova E. (2011). Genome sequencing reveals diversification of virulence factor content and possible host adaptation in distinct subpopulations of *Salmonella enterica*. BMC Genomics.

[137] Sangal V., Harbottle H., Mazzoni C.J., Helmuth R., Guerra B., Didelot X., Paglietti B., Rabsch W., Brisse S., Weill F.X., Roumagnac P., Achtman M. (2010). Evolution and population structure of *Salmonella enterica* serovar Newport. J. Bacteriol..

[138] Holt K.E., Parkhill J., Mazzoni C.J., Roumagnac P., Weill F.-X., Goodhead I., Rance R., Baker S., Maskell D.J., Wain J., Dolecek C., Achtman M., Dougan G. (2008). High-throughput sequencing provides insights into genome variation and evolution in *Salmonella* Typhi. Nat. Genet..

[139] McClelland M., Sanderson K.E., Clifton S.W., Latreille P., Porwollik S., Sabo A., Meyer R., Bieri T., Ozersky P., McLellan M. (2004). Comparison of genome degradation in Paratyphi A and Typhi, human-restricted serovars of *Salmonella enterica* that cause typhoid. Nat. Genet..

[140] Kingsley R.A., Santos R.L., Keestra A.M., Adams L.G., B√§umler A.J. (2002). *Salmonella enterica* serotype Typhimurium ShdA is an outer membrane fibronectin-binding protein that is expressed in the intestine. Mol. Microbiol..

[141] Kingsley R.A., van Amsterdam K., Kramer N., Baumler A.J. (2000). The *shdA* gene is restricted to serotypes of *Salmonella enterica* subspecies I and contributes to efficient and prolonged fecal shedding. Infect. Immun..

[142] Thomson N.R., Clayton D.J., Windhorst D., Vernikos G., Davidson S., Churcher C., Quail M.A., Stevens M., Jones M.A., Watson M. (2008). Comparative genome analysis of *Salmonella* Enteritidis PT4 and *Salmonella* Gallinarum 287/91 provides insights into evolutionary and host adaptation pathways. Genome Res..

[143] Betancor L., Yim L., Fookes M., Martinez A., Thomson N.R., Ivens A., Peters S., Bryant C., Algorta G., Kariuki S., Schelotto F., Maskell D., Dougan G., Chabalgoity J.A. (2009). Genomic and phenotypic variation in epidemic-spanning *Salmonella enterica* serovar Enteritidis isolates. BMC Microbiol..

[144] Fricke W.F., Mammel M.K., McDermott P.F., Tartera C., White D.G., LeClerc J.E., Ravel J., Cebula T.A. (2011). Comparative genomics of 28 *Salmonella enterica* isolates: Evidence for crispr-mediated adaptive sublineage evolution. J. Bacteriol..

[145] Hoffmann M., Zhao S., Luo Y., Li C., Folster J.P., Whichard J., Allard M.W., Brown E.W., McDermott P.F. (2012). Genome sequences of five *Salmonella enterica* serovar Heidelberg isolates associated with a 2011 multistate outbreak in the united states. J. Bacteriol..

[146] Okoro C.K., Kingsley R.A., Quail M.A., Kankwatira A.M., Feasey N.A., Parkhill J., Dougan G., Gordon M.A. (2012). High-resolution single nucleotide polymorphism analysis distinguishes recrudescence and reinfection in recurrent invasive nontyphoidal *Salmonella* Typhimurium disease. Clin. Infect. Dis..

[147] Richardson E.J., Limaye B., Inamdar H., Datta A., Manjari K.S., Pullinger G.D., Thomson N.R., Joshi R.R., Watson M., Stevens M.P. (2011). Genome sequences of *Salmonella enterica* serovar Typhimurium, Choleraesuis, Dublin, and Gallinarum strains of well- defined virulence in food-producing animals. J. Bacteriol..

[148] Feng Y., Liu W.Q., Sanderson K.E., Liu S.L. (2011). Comparison of salmonella genomes. Salmonella: From genome to function.

[149] Leekitcharoenphon P., Lukjancenko O., Friis C., Aarestrup F.M., Ussery D.W. (2012). Genomic variation in *Salmonella enterica* core genes for epidemiological typing. BMC Genomics.

[150] Didelot X., Achtman M., Parkhill J., Thomson N.R., Falush D. (2007). A bimodal pattern of relatedness between the *salmonella* paratyphi a and typhi genomes: Convergence or divergence by homologous recombination?. Genome Res..

[151] Holt K.E., Perkins T.T., Dougan G., Kingsley R.A. (2011). Genomics and pathogenesis of *Salmonella enterica* serovars Typhi and Paratyphi A. Salmonella: From genome to function.

[152] Romling U., Bokranz W., Rabsch W., Zogaj X., Nimtz M., Tschape H. (2003). Occurrence and regulation of the multicellular morphotype in *Salmonella* serovars important in human disease. Int.J. Med. Microbiol..

[153] White A.P., Surette M.G. (2006). Comparative genetics of the rdar morphotype in *Salmonella*. J. Bacteriol..

[154] Mahajan R.K., Khan S.A., Chandel D.S., Kumar N., Hans C., Chaudhry R. (2003). Fatal case of *Salmonella enterica* subsp. *arizonae* gastroenteritis in an infant with microcephaly. J. Clin. Microbiol..

[155] Meric G., Kemsley E.K., Falush D., Saggers E.J., Lucchini S. Phylogenetic distribution of traits associated with plant colonization in *Escherichia coli*. Environ. Microbiol..

[156] Sakellaris H., Hannink N.K., Rajakumar K., Bulach D., Hunt M., Sasakawa C., Adler B. (2000). Curli loci of *Shigella* spp. Infect. Immun..

[157] Bokranz W., Wang X., Tschape H., Romling U. (2005). Expression of cellulose and curli fimbriae by *Escherichia coli* isolated from the gastrointestinal tract. J. Med. Microbiol..

[158] Crawford R.W., Rosales-Reyes R., Ramirez-Aguilar M.d.l.L., Chapa-Azuela O., Alpuche-Aranda C., Gunn J.S. (2010). Gallstones play a significant role in *Salmonella* spp. gallbladder colonization and carriage. Proc. Natl. Acad. Sci. USA.

[159] Lawley T.D., Bouley D.M., Hoy Y.E., Gerke C., Relman D.A., Monack D.M. (2007). Host transmission of *Salmonella enterica* serovar Typhimurium is controlled by virulence factors and indigenous intestinal microbiota. Infect. Immun..

[160] Gordon M.A. (2011). Invasive nontyphoidal *Salmonella* disease. Curr. Opin. Infect. Dis..

